# The effect of distal radius fractures involving the distal radioulnar articular joint on forearm rotation

**DOI:** 10.1186/s13018-020-02091-w

**Published:** 2020-11-19

**Authors:** Lingde Kong, Meng Fu, Jian Lu, Yanqing Zhou, Zuzhuo Zhang, Bing Zhang

**Affiliations:** 1 Department of Orthopedics, The Third Hospital of Hebei Medical University, 139 Ziqiang Road, Shijiazhuang, Hebei 050051 P. R. China; 2 Medical Examination Center, The Third Hospital of Hebei Medical University, Shijiazhuang, Hebei 050051 P. R. China; 3 Department of Radiology, The Third Hospital of Hebei Medical University, Shijiazhuang, Hebei 050051 P. R. China

**Keywords:** Distal radius, Distal radioulnar articular, 3D, Reconstruction, Forearm rotation

## Abstract

**Background:**

The objective of this study was to predict the function of the forearm rotation on the basis of the articular surface of the sigmoid notch from three-dimensional reconstruction images.

**Methods:**

We retrospectively reviewed patients who underwent volar plate fixation for intra-articular distal radius fractures (DRFs) in our institution between January 2017 and July 2019. The 3D image of the sigmoid notch on the fractured distal radius was reconstructed and looked up from the ulnar view to determine the existence of gaps or steps. Patients with or without gaps/steps on the sigmoid notch were included in the case group or control group, respectively. The patients’ basic data and postoperative data were collected and compared.

**Results:**

A total of 81 patients were included. There were 33 patients in the case group, and 48 patients in the control group. There was no significant difference between the two groups at baseline. The total range of motion (ROM) of rotation in the case group and control group was 130.3 ± 6.2° and 145.3 ± 6.7°, respectively (*P* < 0.001). The percentage of rotation ROM of contralateral limb in the case group and control group was 72.3 ± 3.1% and 80.7 ± 3.6%, respectively (*P* < 0.001). VAS during forearm rotation was 2.1 ± 0.7 in the case group, which is significantly higher than that in the control group (1.5 ± 0.5, *P* < 0.001).

**Conclusion:**

This study proposed a new method to assess the articular surface of the sigmoid notch which is based on 3D reconstruction images. With the assistance of this method, we found that gaps or steps on the sigmoid notch not only limit forearm pronation rotation and supination rotation, but also cause apparent wrist pain during forearm rotation movement and poor wrist ability.

## Introduction

Distal radius fractures (DRFs) are the most common fractures of the upper extremity [1, 2]. Although favorable clinical outcomes have been reported after surgical treatment, patients with residual deformity frequently complain of wrist pain, reduced grip strength, and restricted range of motion (ROM) [3, 4]. Wrist flexion and extension frequently attract people’s attention, but the restriction of forearm rotation is often disabling because compensation cannot be provided easily via the shoulder. Normal anatomy of the distal radioulnar joint (DRUJ) comprises the distal radius, ulna, and triangular fibrocartilage complex (TFCC), and the sigmoid notch of the distal radius articulates with the convex ulnar head. Any incongruency of the sigmoid notch and the ulnar head may lead to pain or dysfunction of the DRUJ [5, 6].

For common intra-articular DRFs, X-ray is still the preferred test. Though it is easily performed, X-ray test could only recognize apparent intra-articular fractures and tend to miss small step-offs or gaps, because of the blocking of the ulna and its single projection angle. Computed tomography (CT) images could recognize all small fractures, but it had limited capacity to get a full view of the overall fractures.

Three-dimensional images of the articular surface can be reconstructed by the use of the data transferred from CT. In the 3D reconstruction images, articular surface details such as the fossa, ankle, and destruction of the articular surface were clearly displayed. Compared with traditional CT test, this technique could provide abundant information for surgeons from multiple angles [7, 8].

Though the 3D reconstruction technique was frequently used to assist orthopedic surgery, it has never been used to assess the articular surface of the sigmoid notch. The objective of this study was to confirm if the 3D reconstruction images could provide additional information for us to predict the function of forearm rotation, especially in patients with intra-articular DRFs.

## Materials and methods

### Study design and patient population

We retrospectively reviewed patients who underwent volar plate fixation for intra-articular DRFs in our institution between January 2017 and July 2019. The inclusion criteria were patients over 18 years old with intra-articular DRFs confirmed by X-ray test or CT examination. Patients with open fractures, associated carpal bone fractures, radial head injury, dislocation of the distal radioulnar joint, injuries of the TFCC, or history of the hand or upper extremity surgery were excluded from this study. Patients with incomplete follow-up data were also excluded. This study was approved by the Research and Ethics Committee of the Third Hospital of Hebei Medical University, and all patients gave written informed consent for their information to be stored in the database of this hospital and used for medical research.

### Treatment and follow-up

All patients underwent open reduction and volar plate fixation with brachial plexus or general anesthesia. The locking plate is applied through an incision over the volar aspect of the wrist, and no combined dorsal approach is used. All surgeries were performed by three surgeons. The details of the surgical approach, the type of plate, and the number and configuration of screws were decided by the surgeons. Some surgeons used a cast/splint after the procedure, but the fixed angle stability provided by the locking plate is generally sufficient to allow an early controlled range of movement exercises. The use or otherwise of a cast/splint was also at the discretion of the surgeon. The finger, elbow, and shoulder exercises were started on the first day after surgery. After surgery, an additional CT examination was performed and analyzed.

Patients were followed-up at 4 weeks, 6 weeks, and then every 2 weeks until fracture healing. Wrist exercise advice was given by surgeons according to the status of fracture healing, which is a standard post-operative rehabilitation protocol including six wrist movements at a certain frequency. At the 1-year follow-up, the additional clinical assessment was performed. ROM of bilateral forearm rotation was measured using a goniometer, and pronation as well as supination motion was measured based on a neutral position. Pain in forearm rotation was evaluated by visual analog scale (VAS), with 0 representing no pain and 10 representing maximum pain. The wrist ability was assessed by the Patient-Rated Wrist Evaluation (PRWE).

### Data collection and parameter evaluation

The patients’ basic data were collected, including the age, gender, osteoporosis, dominant extremity fracture, preoperative swelling, AO classification, associated ulnar styloid process fracture, and time from injury to operation. Surgical-related data were also collected, which included additional Kirschner wire fixation, post-operative radial inclination, post-operative volar tilt, post-operative ulnar variance, removal of the volar plate, and assisted cast or splint fixation. Preoperative swelling was assessed on the first day of hospitalization. If the wrist is swelling than the contralateral side but the skin texture can be recognized, the swelling was considered to be slight. If the skin texture cannot be recognized or blisters occurred, the swelling was considered to be severe. Measurements for the ulnar inclination, volar angulation, and ulnar variance were performed on the postoperative radiographs according to the previous method [4].

**Fig. 1:**
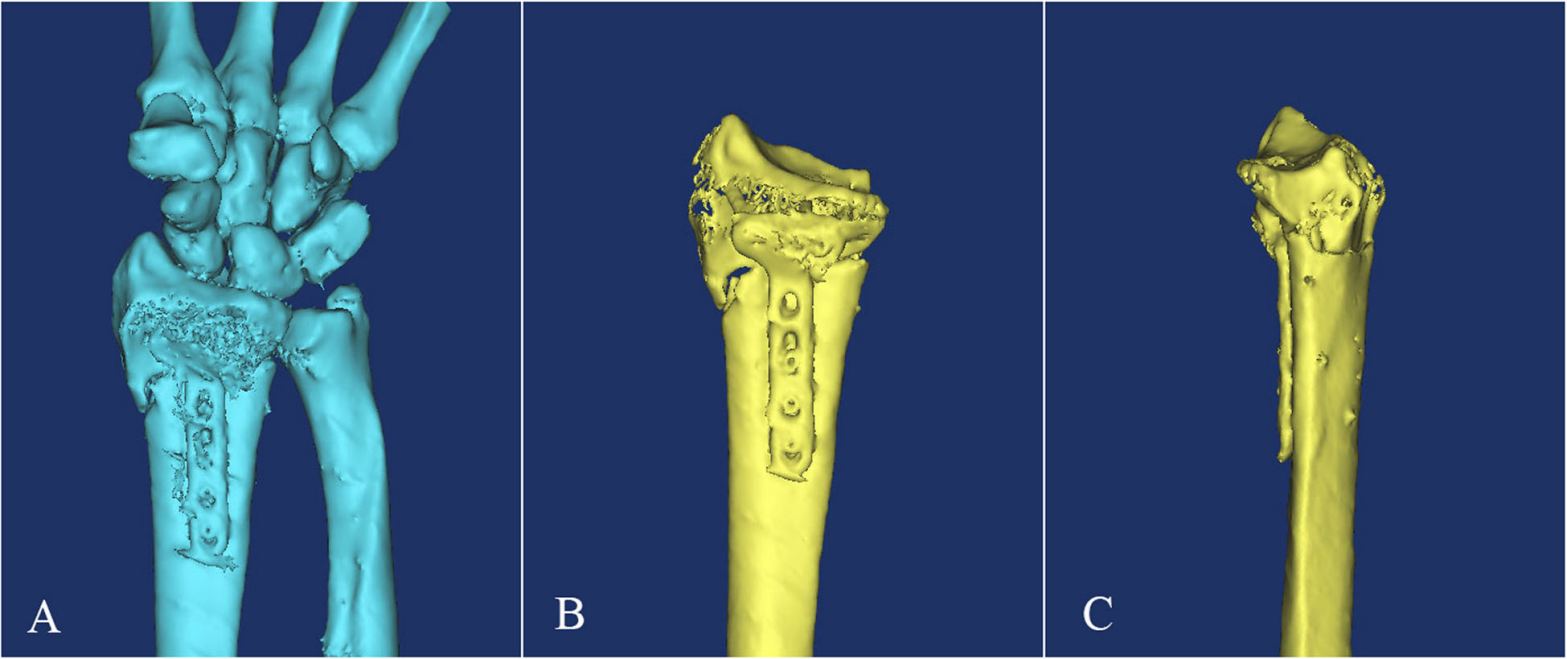
The 3D reconstruction images of the distal radius. **a** The wrist joint was reconstructed. **b** The distal radius was separated from the carpal bone and ulnar bone. **c** The 3D reconstruction image of the sigmoid notch was shown

The patients after surgery were required to perform a 1.0-mm CT scan (Aquilion 64; Toshiba, Tokyo, Japan) of the distal radius, and the data were kept in the DICOM format and were 3D reconstructed using Mimics20.0 (Materialise, Belgium). The editing function of software was used to separate the distal radius from the carpal bone and remove the ulnar bone image. The 3D reconstruction image of the sigmoid notch on a fractured distal radius was looked up from the ulnar view to determine the existence of gaps or steps (Fig. 1). Gaps or steps were considered when the distance of fracture borders was more than 1 mm. Patients with gaps or steps on the sigmoid notch were included in the case group; otherwise, they were included in the control group.

### Statistical analysis

Data analyses were performed using SPSS version 20 for Windows (SPSS, Inc., Chica go, IL, USA). Data are presented as the number of subjects in each group or mean ± standard deviation (SD). To determine the difference between groups, Fisher’s exact tests or independent-samples *t* tests were used. A probability value of less than 0.05 was considered statistically significant.

## Results

A total of 81 patients were included in our study. Among them, 33 patients with unsmooth sigmoid notch were enrolled in the case group, and the other 48 patients were enrolled in the control group. The age of patients in the case group and control group were 50.9 ± 6.1 years and 52.2 ± 5.3 years, respectively. Severe preoperative swelling was shown in 14 and 22 patients in the case group and control group, respectively. The time from injury to operation in the two groups was 3.8 ± 2.1 days and 3.7 ± 2.4 days, respectively. There was no apparent loss of reduction or intra-articular screw penetration in both groups. Preoperative basic data and postoperative radiographic parameters were listed in Table 1. There was no significant difference between the two groups at baseline.

**Table 1:** The basic data of patients with intra-articular distal radius fractures

	Case group	Control group	*P* value
No. of patients	33	48	
Age (years)	50.9 ± 6.1	52.2 ± 5.3	0.311
Gender (male/female)	15/18	20/28	0.821
Osteoporosis (yes/no)	12/21	19/29	0.819
Dominant extremity fracture (yes/no)	17/16	28/20	0.650
Preoperative swelling (slight/severe)	19/14	26/22	0.822
AO classification (type B/type C)	9/24	11/37	0.794
Associated ulnar styloid process fracture (yes/no)	11/22	18/30	0.815
Time from injury to operation (days)	3.8 ± 2.1	3.7 ± 2.4	0.847
Additional Kirschner wire fixation (yes/no)	20/13	24/24	0.373
Post-operative radial inclination (degree)	20.2 ± 3.4	21.1 ± 3.1	0.221
Post-operative volar tilt (degree)	6.5 ± 2.3	7.1 ± 2.7	0.300
Post-operative ulnar variance (mm)	2.3 ± 0.8	2.2 ± 0.5	0.491
Removal of volar plate (yes/no)	13/20	17/31	0.816
Assisted cast or splint fixation (yes/no)	18/15	22/26	0.502

**Fig. 2:**
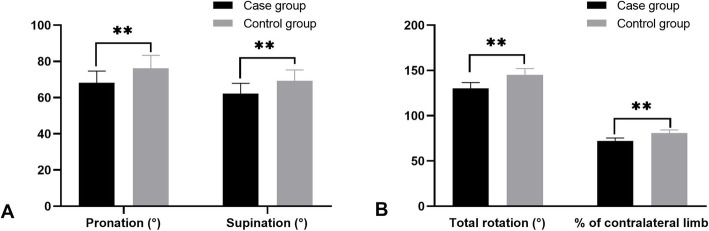
The histogram showing the function results of forearm rotation. **a** The results of forearm pronation and supination. **b** The results of the total rotation and percentage of the contralateral limb. **P < 0.001

At the final follow-up, ROM of pronation was 68.2 ± 6.4° in the case group, which is significantly lower than that in the control group (76.1 ± 7.3°, *P* < 0.001). Similarly, the ROM of supination was 62.1 ± 5.8° in the case group, which is significantly lower than that in the control group (69.2 ± 6.1°, *P* < 0.001). Total ROM of rotation in the case group and control group were 130.3 ± 6.2° and 145.3 ± 6.7°, respectively, and the difference showed statistically significant (*P* < 0.001). The percentage of rotation ROM of the contralateral limb in the case group and control group were 72.3 ± 3.1% and 80.7 ± 3.6%, respectively, and the difference showed statistically significant (*P* < 0.001). The details were shown in Fig. 2. VAS during forearm rotation were 2.1 ± 0.7 in the case group, which is significantly higher than that in the control group (1.5 ± 0.5, *P* < 0.001). The PRWE score in the case group and control group were 24.5 ± 6.8° and 19.4 ± 5.2°, respectively, and the difference showed a statistically significant (*P* < 0.001).

## Discussion

The sigmoid notch of the radius is rarely studied because of its hidden position. In the current study, the sigmoid notch was clearly displayed by the use of the 3D reconstruction technique with data transferred from postoperative CT test, and corresponding details help to predict postoperative rotation function and pain score during activity. The results showed that patients with gaps or steps on the sigmoid notch not only limit forearm pronation rotation as well as supination rotation, but also cause apparent wrist pain during forearm rotation movement and poor wrist ability.

Three-dimensional reconstruction technology has been rapidly developed in the medical field and has become feasible and accessible for wild applications in orthopedic surgery [9–11]. It is extremely helpful for preoperative evaluation and planning as well as for intraoperative navigation, but few studies used this technique to predict the outcomes of DRUJ after surgical treatment of DRFs. After the CT images are transformed into 3D images, the occlusion of other bones can be well solved. The ulna and carpal bones are easily removed, and thus, the surface of the sigmoid notch can be clearly displayed, which makes the following measurement, classification, and further analysis become feasible [12].

Free forearm rotation relies on a normal structure of the proximal radioulnar joint as well as the distal radioulnar joint. DRUJ comprises the distal radius, ulna, and triangular fibrocartilage complex. Malunited DRFs were frequently found to cause forearm rotational restriction, for example, previous studies have revealed that a dorsal angulated deformity of more than 30° causes pronation restriction and a volar angulated deformity of more than 20° causes supination restriction [13, 14]. Radial shortening of 10 mm also causes rotational restriction [15]. However, the association of contact areas with forearm rotation is rarely investigated. The sigmoid notch not only serves as an anchor for the TFCC that plays an important role in DRUJ stability, but also provides a smooth articular surface for a rotation movement. When the fracture line or displaced fragment of the distal radius involving the sigmoid notch, the tension of the TFCC may be changed, which would produce a rotation dysfunction of the forearm [5, 16]. Besides, the gaps or steps could cause articular incongruity of the DRUJ and limited forearm rotation. This could explain our final result that both the pronation and supination rotation was restricted in patients with a fractured sigmoid notch in comparison with those without.

Postoperative pain is common in DRF patients even after surgical treatment [17]. The irregular articular surface has been demonstrated to be the main reason to increase the risk of traumatic osteoarthritis and to cause unsatisfactory function recovery [18–20], and if it is associated with postoperative pain remains unclear. To clarify this confusion, we made a comparison, and the results showed that patients with gaps or steps on the sigmoid notch have significantly more severe pain during forearm rotation at the 1-year follow-up. We supposed that postoperative pain symptoms begin much earlier before osteoarthritis develops. This information allows doctors to have a clearer understanding of the prognosis of fracture and to better communicate with patients.

This study has several limitations. First of all, this is a retrospective study. The study design and potential for bias are the typical restrictions of our study. Secondly, the exact degree of reduction by surgery which left minimized rotation dysfunction has not been established. Thirdly, limited by the accuracy of the CT test, the current technology is unable to differentiate tiny fracture fragments, especially in patients with severely comminuted fractures. Finally, the follow-up time is relatively short, which is only 1 year. Wrist degenerative changes or osteoarthritis may occur in a long-term follow-up and should be recorded in further studies.

## Conclusions

This study proposed a new method to assess the articular surface of the sigmoid notch which is based on three-dimensional reconstruction images. With the assistance of this method, we found that gaps or steps on the sigmoid notch not only limit forearm pronation rotation and supination rotation, but also cause apparent wrist pain during forearm rotation movement and poor wrist ability. This information help doctors to have a better understanding of the prognosis of intra-articular DRFs.

## Data Availability

Not applicable.

## Abbreviations

DRFs: Distal radius fractures
ROM: Range of motion
DRUJ: Distal radioulnar joint
TFCC: Triangular fibrocartilage complex
CT: Computed tomography
VAS: Visual analog scale
SD: Standard deviation
